# p53 Calls upon CIA (Calcium Induced Apoptosis) to Counter Stress

**DOI:** 10.3389/fonc.2015.00057

**Published:** 2015-03-10

**Authors:** Sue Haupt, Dinesh Raghu, Ygal Haupt

**Affiliations:** ^1^Tumour Suppression Laboratory, Peter MacCallum Cancer Centre, Melbourne, VIC, Australia; ^2^Sir Peter MacCallum Department of Oncology, The University of Melbourne, Parkville, VIC, Australia; ^3^Department of Pathology, The University of Melbourne, Parkville, VIC, Australia; ^4^Department of Biochemistry and Molecular Biology, Monash University, Melbourne, VIC, Australia

**Keywords:** p53, SERCA, apoptosis, mitochondria, endoplasmic reticulum

The tumor suppressive function of p53 is a fundamental barrier against cancer development. This is attested by the high frequency of p53 mutations in human cancer, more than any other known gene. Therefore, the mechanisms by which p53 suppresses cancer have attracted much attention. These can be broadly divided into functions that inhibit the growth and propagation of cancer or transformed cells (growth inhibitory activities), and functions that prevent cells from becoming cancerous [reviewed in Ref. (1)]. The mechanisms underlying tumor prevention by p53 are poorly defined and have recently attracted a new wave of interest [reviewed in Ref. (2)]. On the other hand, much more is understood about the mechanisms by which p53 eliminates cancer cells. In response to oncogenic stress, as well as other stress conditions, p53 triggers growth inhibitory functions, including growth arrest, senescence, autophagy, and apoptosis (1, 2). p53 triggers these cellular responses by inducing the expression of a large number of target genes.

While the transcriptional activity of p53 is important for its apoptotic function, an early study provided vital direct indication that a transcriptional deficient p53 mutant is able to induce cell death (3). This report provided the rationale for investigating the transcriptional-independent apoptotic activities of p53. Over the past two decades, the cytoplasmic tumor suppressive functions of p53 have been studied, revealing a role for cytoplasmic p53 in mitochondrial apoptosis and in the suppression of autophagy, which acts as a survival mechanism under metabolic stress conditions [reviewed in Ref. (4)]. It is important to note that both processes are also controlled by nuclear p53: the mitochondrial apoptosis through the powerful induction of Puma, Bax, and Noxa; and autophagy by the induction of genes such as DRAM.

Giorgi and co-workers recently described an important addition to our understanding of the cytoplasmic apoptotic function of p53. In their studies published in PNAS (5) and Oncotarget (6), they utilized novel calcium imaging tools to provide a new and timely interpretation of how cytoplasmic p53 provokes cell death. They show that in response to stresses, wild type (wt) p53 accumulates at the endoplasmic reticulum–mitochondrial-associated membranes (ER–MAMs), where it interacts with sarco-endoplasmic reticulum Ca^2+^ ATPase (SERCA) pump to promote calcium overload in the mitochondria. In turn, this drives the loss of membrane potential and culminates in apoptosis (Figure 1). This novel finding provides a vital fragment of clarification for the p53 conundrum regarding its functions independent of its transcriptional activity.

**Figure 1:**
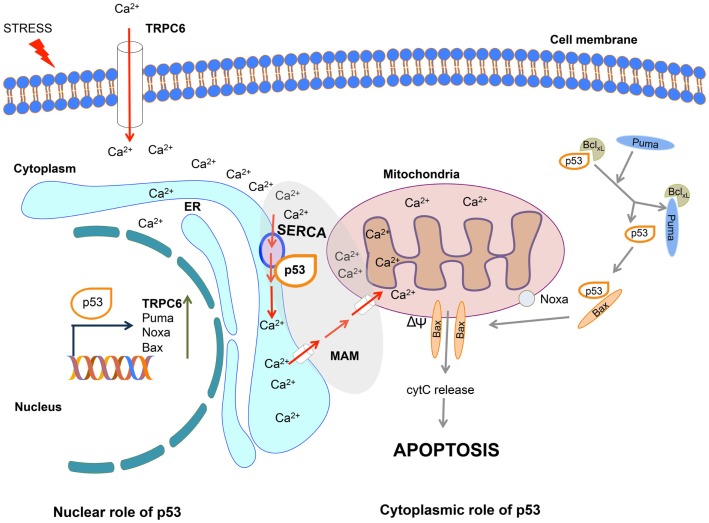
**Nuclear and cytoplasmic role of p53 in the induction of calcium-induced apoptosis**. In response to stress, nuclear p53 activates the transcription of pro-apoptotic genes including TRPC6, Puma, Noxa, and Bax. Cytoplasmic p53 interacts with SERCA at the endoplasmic reticulum (ER)/mitochondrial-associated membrane (MAM) junction, leading to the increase in Ca^2+^ influx, thereby promoting the loss of membrane potential (ΔΨ), release of cytochrome *c*, and induction of apoptosis. The entry of calcium into the cell is further enhanced through TRPC6 channel. Through this combined nuclear and cytoplasmic actions p53 promotes calcium-mediated apoptosis. For simplicity, the other apoptotic functions of p53 are not depicted in this diagram.

Linking p53, the pivotal orchestrator of cellular stress responses to calcium (Ca^2+^), which is a vital transducer of cell death, ties together two fundamental dictators of apoptosis. Genotoxic damage and oxidative stress are both effective stimuli of p53-driven calcium activation. Ca^2+^ concentration is critical for its functions (including its wider role as a vital secondary messenger in cell signaling), which defines the importance of calcium pumps to generate gradients across membranes. These findings predict that the p53–SERCA connection would not be functional in cancer cells. Consistent with this prediction, SERCA expression is frequently decreased or lost in colon carcinomas and is low in colon and gastric cell lines (7). Similarly, the p53–SERCA function is compromised in breast cancer, where SERCA expression is lost with increased tumor aggressiveness [Ref. (8); reviewed in Ref. (9)]. Importantly, Giorgi et al. (6) showed that the interaction between p53 and SERCA is lost when p53 is mutated. These studies demonstrate the importance of deregulating the p53–SERCA functions during cancer progression either by depleting SERCA or selecting for p53 mutations. It will also be interesting to examine whether the p53 SNP72 impacts on the efficiency of SERCA-mediated apoptosis, where codon 72 Arg was shown to be more prone to mitochondrial apoptosis than codon 72 Pro [reviewed in Ref. (10)]. Significantly, therapeutic targeting of calcium pumps is being investigated [reviewed in Ref. (9)]. For example, SERCA inhibitory agents are considered for the treatment of T-ALL (11). The findings by Giorgi et al. argue that in cancers where apoptosis depends on p53–SERCA, such therapeutic approaches may be debilitating.

While the studies by Giorgi et al. (5) using a p53 mutant lacking the nuclear localization signal, define a nuclear/transcriptional-independent role for p53 at the ER-mitochondria, p53 has links to calcium beyond this critical function. p53 has recently been demonstrated to transcriptionally activate the calcium channel, transient receptor potential cation channel subfamily C member 6 (TRPC6), in response to the anti-cancer drug derivative of gallium. TRPC6 mediates Ca^2+^ cellular influx across the plasma membrane (12). Together these fascinating findings implicate calcium to be instrumental in the p53 response both in (1) a transcriptionally dependent manner through elevation of TRPC6 levels to increase cytoplasmic Ca^2+^ levels and (2) a transcriptionally independent mechanism through its interaction with SERCA driving Ca^2+^ from the ER into the mitochondria, with a consequent loss of membrane potential. Thus, p53 is pivotal in both priming and executing cell stress-driven apoptosis in a Ca^2+^-dependent manner. Once again, as in the case of the mitochondrial apoptotic function of p53 directed through the BH-domain proteins [Puma, Noxa, and Bax reviewed in Ref. (4); Figure 1], here too p53 employs multiple arms to augment the response, and also possibly as a safe guard mechanism. It would be interesting to define the extent of the p53–SERCA apoptotic function in normal cells, during development and whether it is cell type dependent.

## Conflict of Interest Statement

The authors declare that the research was conducted in the absence of any commercial or financial relationships that could be construed as a potential conflict of interest.
